# Flow-cytometry analysis reveals persister resuscitation characteristics

**DOI:** 10.1186/s12866-020-01888-3

**Published:** 2020-07-08

**Authors:** Sayed Golam Mohiuddin, Pouria Kavousi, Mehmet A. Orman

**Affiliations:** Department of Chemical and Biomolecular Engineering, University of Houston, S222 Engineering Bldg 1, 4726 Calhoun Rd, Houston, TX 77204 USA

**Keywords:** *Escherichia coli*, Flow cytometry, Persister resuscitation, Viable but non-culturable cells, ATP depletion, Beta-lactams, Fluorescent proteins, Single-cell analysis, Arsenate pretreatment

## Abstract

**Background:**

Persisters and viable but non-culturable (VBNC) cells are two phenotypic variants known to be highly tolerant to antibiotics. Although both cell types are stained as live and often appear as nongrowing during antibiotic treatment, the only distinguishing feature is the ability of persisters to recolonize in standard culture media in the absence of antibiotics. Despite considerable progress in the characterization of persister formation mechanisms, their resuscitation mechanisms remain unclear due to technical limitations in detecting and isolating these cell types in culture environments that are highly heterogeneous.

**Results:**

In this study, we used a methodology integrating flow cytometry, fluorescent protein expression systems and ampicillin-mediated cell lysing technique to monitor persister resuscitation at the single-cell level. With this method, we were able to investigate the effects of various culture conditions (e.g., antibiotic treatment time, the length of the stationary phase in overnight pre-cultures, or pretreatment of cells with a metabolic inhibitor) on persister resuscitation. Although we observed long-term pre-cultures have many more VBNC cells compared to short-term pre-cultures, only a small fraction of non-lysed cells was able to resuscitate in all conditions tested. Regardless of pre-culturing and ampicillin treatment times, these persister cells started to resuscitate within 1 hour, after they were transferred to fresh liquid media, with the same doubling time that normal cells have. Our analysis further showed that ampicillin was not able to lyse the cells in the presence of arsenate, a metabolic inhibitor commonly used to increase bacterial persistence. However, the removal of arsenate during antibiotic treatment resulted in cell lysis and a reduction in persister levels despite the significant decrease in ATP levels in the cells.

**Conclusions:**

The strategy presented in this study helps us monitor persister resuscitation at the single-cell level, and simultaneously quantify persister, VBNC and dead cell subpopulations in ampicillin-treated cultures. Our results indicate that the characterization of persister resuscitation with flow cytometry will enhance the current molecular-level understanding of persistence and its evolution.

## Background

Persistence is a non-genetic and non-heritable bet-hedging strategy that has evolved in many prokaryotic and eukaryotic cell types [1]. It results from a phenotypic switch from a normal, growing state to a tolerant, non-growing state. Environmental heterogeneity is thought to produce selection pressures that maintain the persister phenotypes. Both stochastic processes and deterministic factors, pertaining to stress responses, cell age, quorum sensing and toxin/antitoxin systems, are known to facilitate persister formation [1–4]. Regardless of the extended periods of latency, persister cells have the ability to exit from the persistence state and establish heterogeneous cell populations.

Despite the considerable progress in the characterization of persister formation mechanisms, the physiology of these phenotypic variants remains unclear. The assumption that persisters are globally dormant cells [5, 6] has discouraged many scientists from characterizing their physiology. Moreover, direct assessments of persister physiology using conventional approaches, such as mass spectrometry or transcriptomic profiling, are currently hindered by limitations in the isolation procedures. Despite their notable contributions to persister research, existing strategies [7–11] often yield samples that are enriched in persisters but are highly contaminated with other cell types, such as viable but non-culturable cells (VBNCs), which are generally more abundant than persisters (~ 2-log-fold more) [8, 9]. Although persister and VBNC cells are concurrently present in cell cultures, appear as non-growing and stay alive during antibiotic treatments, the only distinguishing feature is the ability of persisters to recolonize in standard culture media in the absence of antibiotics whereas the resuscitation of VBNCs on such media is rarely possible [12, 13]. However, certain environments, such as plating on media with the antioxidants and catalases, have been shown to restore VBNC cell culturability [14]. It is possible that persistence represents a transitory phase leading to the VBNC state and contributes to the accumulation of VBNC cells due to the accumulation of stress-induced intracellular damage.

The phenotypic features that allow persister resuscitation, a hallmark of persistence, remain relatively unexplored; we still don’t know what mechanisms trigger these phenotypic variants to exit from the persistent state, to repopulate upon cessation of antibiotic treatment, and to regain antimicrobial sensitivity. Unfortunately, detecting and isolating resuscitating persisters in antibiotic-treated cultures is challenging due to their low abundance in a highly heterogeneous environment that also contains dead cells, debris, and VBNC cells [12]. Here, we have overcome these technical limitations by using a methodology that integrates various techniques (e.g., cell division and antibiotic tolerance assays, and flow cytometry analysis) in a tailored approach. This method, which has enabled us to monitor persister resuscitation as well as quantify the persister- and VBNC-cell fractions, will enhance our current understanding of persister resuscitation processes.

## Results

### Monitoring the resuscitating cells with the protein-dilution method

**Fig. 1:**
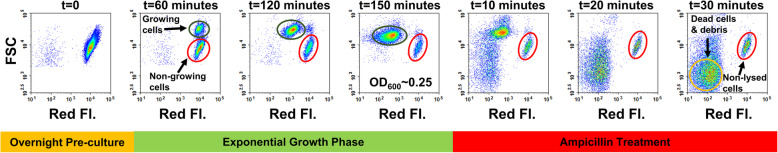
Isolating non-growing cell subpopulations with ampicillin-induced cell lysing and protein-dilution methods. mCherry positive cells from overnight (24 h) pre-cultures were diluted 100-fold in fresh LB broth without IPTG. Upon reaching the exponential-growth phase (OD_600_ = 0.25), cells were treated with ampicillin at 10X MIC concentration (60 μg/ml). Growing cell, non-growing cell and dead-cell/debris subpopulations are highlighted with dark green, red and orange circles, respectively. Red Fl.: Red Fluorescence

One of the common persister-isolation techniques involves treating cultures with beta-lactams (such as ampicillin) and sedimentation of the non-lysed cell population [10]. Ampicillin inhibits transpeptidase enzymes involved in bacterial cell wall synthesis, thus targeting proliferating cells only [15, 16]. This was also verified by a fluorescent protein-dilution method here (Fig. 1), a strategy commonly used to monitor cell growth [3, 9]. In this method, we first induced *mCherry* expression during overnight growth of an *E. coli* strain that harbors a chromosomally integrated IPTG-inducible mCherry expression cassette. The mCherry-positive cells from the overnight pre-culture (Fig. 1, t = 0) were then inoculated into a fresh medium without the inducer. At time zero, all cells exhibited a high level of mCherry (red) fluorescence, which declined as the cells divided, except in a small subpopulation (~ 4% of the entire population at t = 150 min, OD_600_ = 0.25) whose fluorescence remained constant due to the lack of division (Fig. 1, subpopulations highlighted with red circles). As expected, the growing cells, exhibiting higher forward scatter (FSC) signals, became filamented and were lysed rapidly upon exposed to ampicillin; however, the non-growing cell population, which was shown to be enriched with persister and VBNC cells [8, 12], remained intact (Fig. 1).

**Fig. 2:**
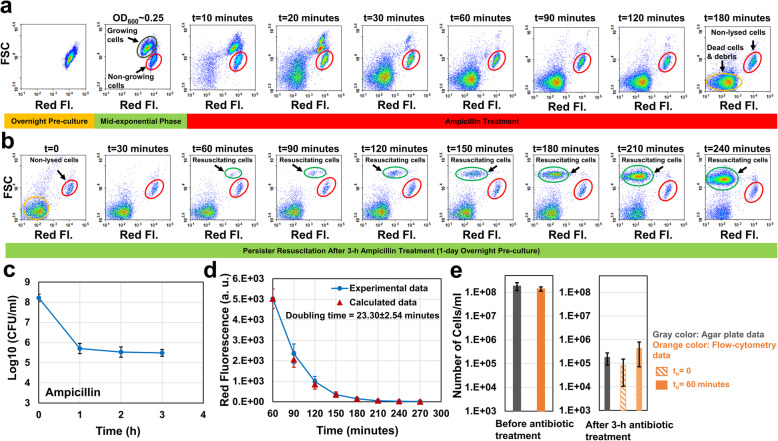
Monitoring persister resuscitation. **a** Exponential-phase cells at OD_600_ = 0.25 (prepared from 1-day overnight pre-cultures) were treated with 60 μg/ml ampicillin for 3 h in the presence of IPTG. Cells during the treatment were collected at designated time points and analyzed by a flow cytometer. **b** After 3-h ampicillin treatments, cells were collected and washed to remove the antibiotic and the inducer. The cells were then resuspended into fresh LB broth and cultured. At designated time points, samples were collected to be analyzed with a flow cytometer to monitor persister resuscitation (*N* = 4). **c** Cells during the antibiotic treatment were plated on agar media at designated time points for CFU enumeration (*N* = 4). **d** The doubling time of the resuscitating cells was calculated using the decay equation (see the Materials and Methods) and the mean red fluorescence intensities of dividing cells (highlighted with light green circles) (*N* = 4). **e** Resuscitating cell levels (number of cells per 1 ml culture medium) after 3-h antibiotic treatments were estimated from the standard agar plating method (gray column) and the flow-cytometry analysis (assuming t_o_ = 0 and t_o_ = 60 min; patterned orange columns). Cells were also counted before the antibiotic treatments. Growing, non-lysed, dead (debris) and resuscitating cell subpopulations are highlighted with dark green, red, orange and light green circles, respectively. The flow-cytometry diagrams are a representative replicate of four independent biological replicates. Error bars represent the standard deviations

Using the protein dilution and ampicillin-induced cell lysing techniques, we wanted to monitor persister resuscitation on flow-cytometry diagrams. Unlike the strategy described above, IPTG was kept in the media during the exponential growth phase and ampicillin treatment. Although persisters are largely assumed to be pre-existing non-growing cells, antibiotics are also known to induce cell dormancy and persistence in proliferating cells [17]. In fact, up to 20% of persister cells can arise from growing cell subpopulations [8]. Therefore, IPTG was used to maintain high fluorescent signals in these persister types. When we treated the mid-exponential-phase cells (OD_600_ = 0.25, Fig. 2a) with ampicillin, the growing cells eventually lost their membrane integrity and mCherry (Fig. 2a, t = 10 to 180 min), as expected. In contrast, live, intact cells, comprising persister and VBNC cells, retained high fluorescence (Fig. 2a, t = 180 min, the subpopulation highlighted with a red circle). Our flow cytometry images showed that a 3-h treatment is sufficient to lyse all antibiotic sensitive cells (Fig. 2a, t = 180 min). This treatment length was also found to be sufficient to obtain a bi-phasic kill curve of colony-forming unit (CFU) counts, which ensures the enrichment of persisters and the death of non-persister cells in the cultures (Fig. 2c). After the treatment, cells were washed to remove the antibiotic and IPTG, and then transferred to fresh Luria-Bertani (LB) broth to stimulate persister resuscitation. Persisters, unlike VBNCs, can exit from their non-proliferating phenotypic state and proliferate upon removal of antibiotics. The resuscitating cells were detected by monitoring single-cell mCherry levels using a flow cytometer. We observed that, although the surviving live cells initially exhibited high fluorescence, upon resuscitation in the absence of IPTG, flow cytometry revealed ongoing cell division as the dilution of mCherry protein (Fig. 2b, subpopulations highlighted with green circles). Forward scatter was also expected to increase due to the elongation characteristic of the growing cells. The fluorescence of the cells that did not resuscitate (i.e., VBNCs) remained constant due to lack of cell division (Fig. 2b, subpopulations highlighted with red circles).

### Ampicillin persisters wakeup within one hour

Our flow-cytometry data indicates that persister cells started to resuscitate within 1 hour upon their transfer to fresh media (Fig. 2b). We observed a similar trend in the control cultures where stationary-phase cells from overnight pre-cultures were transferred to fresh media without receiving antibiotic treatments. These untreated cells also started to divide within 1 hour (Additional file 1: Fig. S1A). The doubling time of resuscitating persisters (estimated from the fluorescence decay equation; see Methods) was obtained as 23.30 ± 2.54 min (Fig. 2d), which was found to be consistent with the doubling time of normal, untreated cells (23.91 ± 1.7 min) that grew in LB (Additional file 1: Fig. S1B). We also estimated the initial number of persister cells (N_o_) that woke up in fresh liquid cultures, using the classical exponential-growth equation, $N={N}_o{2}^{\left(t-{t}_o\right)/{t}_d}$, where t_d_ is the doubling time (calculated from the fluorescence decay equation) and N is the number of resuscitated cells at time t. For N, we used the flow cytometry cell counts obtained at t = 240 min (Fig. 2b, highlighted with a green circle). Although it is hard to predict the exact initial resuscitation time (t_o_) of persister cells, our flow-cytometry diagrams showed that these cell subpopulations became visible at t = 60 min (Fig. 2b, highlighted with a green circle), indicating that their wake-up time (t_o_) should be less than 60 min. The same trend was also observed during the wake-up of untreated, stationary phase cells (Additional file 1: Fig. S1A). Here, we used two scenarios to calculate the N_o_ levels from the flow-cytometry diagrams: (i) assuming t_o_ = 0, a scenario that underestimates N_o_ levels since resuscitation is not expected to happen at this time point, or (ii) assuming t_o_ = 60 min, a scenario that overestimates N_o_ levels since persister cells have already resuscitated (Fig. 2e, patterned orange columns). The actual N_o_ should be between these two calculated values. Since the resuscitating cells at 60 min were measurable by the flow cytometer, we compared the calculated cell levels at t_o_ = 60 min with the experimentally determined values to further validate our computational analysis. As expected, we did not see a significant difference between the calculated and experimental data (Additional file 1: Fig. S2A). We also quantify N_o_ levels using the standard agar plating method; briefly, samples after ampicillin treatments were collected, washed to dilute antibiotics to sub-MIC levels, and plated on agar plates. Once a persister cell starts dividing, it forms a colony; thus, the CFU levels correlate with the number of resuscitating cells on agar plates. N_o_ estimates from the flow-cytometry analysis and the CFU measurements of agar plates were found to be consistent and within the expected range (Fig. 2e). Still, resuscitating cells, as measured by CFUs, were calculated as ~ 4% of the non-lysed cell subpopulation. The remaining, ~ 96% cells, classified as VBNCs, did not resume replication. Consistent with the previously published results [8, 9, 12], VBNC cells were found to be more abundant than persisters in our cultures as well. We note that the dilution of mCherry in resuscitating cells is not due to the protein leakage caused by compromised membranes. In order to verify that these cells are alive, we used a pQE-80 L expression system, enabling us to tightly regulate the expression of a green fluorescent protein (GFP) with IPTG. Unlike the mCherry-dilution method, GFP was not induced initially. Cells were first treated with ampicillin for 3 h in the absence of the inducer, and then, transferred to fresh media with IPTG. As expected, a similar fraction of cell subpopulation started to resuscitate and overexpress GFP (Additional file 1: Fig. S3ABC), verifying that resuscitating cells are live cells with active metabolic mechanisms.

### Long-term ampicillin treatment did not affect the persister resuscitation and doubling time

**Fig. 3:**
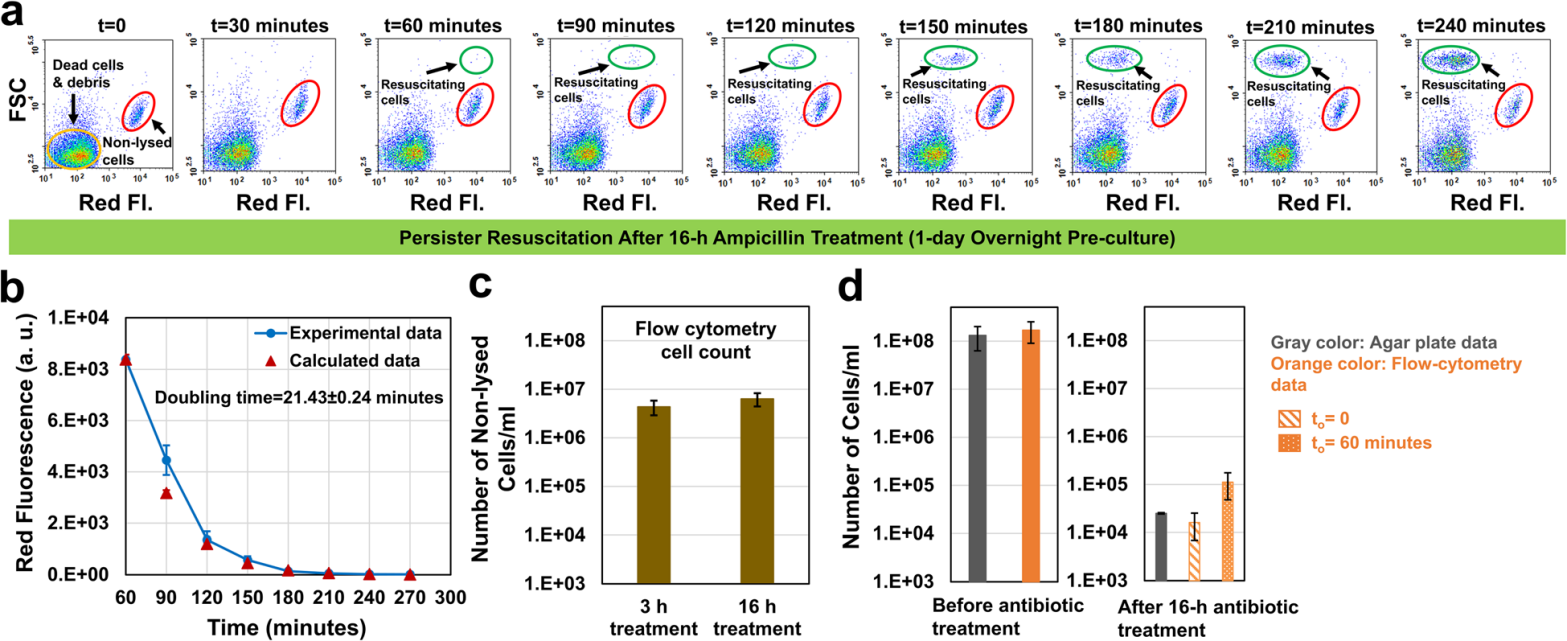
Effect of long-term antibiotic treatment on persister resuscitation. **a** After 16-h ampicillin treatment, cells were transferred to fresh LB broth to monitor persister resuscitation with a flow cytometer (*N* = 4). **b** The doubling time of the resuscitating cells (*N* = 4). **c** Comparison of non-lysed cell levels in short-term and long-term ampicillin treated cultures. Cells were counted with flow cytometry (*N* = 4). **d** Cell counts before antibiotic treatments and resuscitating cell levels after 16-h antibiotic treatments (estimated from agar plates or flow-cytometry analysis) (*N* = 4). Error bars represent the standard deviations

To elucidate the effect of long-term antibiotic treatments on persister resuscitation, we treated the mid-exponential-phase cells with ampicillin for 16 h. Although, non-lysed cell levels quantified by flow cytometry in short- and long-term persister assay cultures (3-h treatment vs. 16-h treatment) were found to be similar (Fig. 3c), persister levels obtained from CFU measurements were slightly lower (*P* < 0.05) in 16-h treatment cultures (Figs. 2e and 3d; gray columns representing the 3-h or 16-h ampicillin treatments). Since the slower-killing phase in the biphasic kill curves is the hallmark of the persistence phenotype, it is possible that longer exposure to the antibiotic has killed more persister cells or converted some of them into VBNC cells. However, the observed increase in VBNC levels in the long-term persister assay cultures is not statistically significant (Additional file 1: Fig. S4), which may be expected considering that VBNCs are orders of magnitude more abundant than persisters.

Persister cells similarly started to resuscitate in fresh LB broth within 60 min after the removal of antibiotics (Fig. 3a, t = 60 min, the subpopulation highlighted with a green circle) with a doubling time (21.43 ± 0.24) (Fig. 3b) similar to that of untreated cells (Additional file 1: Fig. S1B). CFU measurements from agar plates were found to be within the range of N_o_ levels estimated from our flow-cytometry analysis (Fig. 3d and Additional file 1: Fig. S2B), although persister cells still constitute a small fraction of the non-lysed cell subpopulation. Overall, these results unveiled that long-term treatments do not pose any impact on non-lysed cell levels as well as persister-wake up time or doubling time.

### Long-term pre-culturing enhances VBNC cell levels

**Fig. 4:**
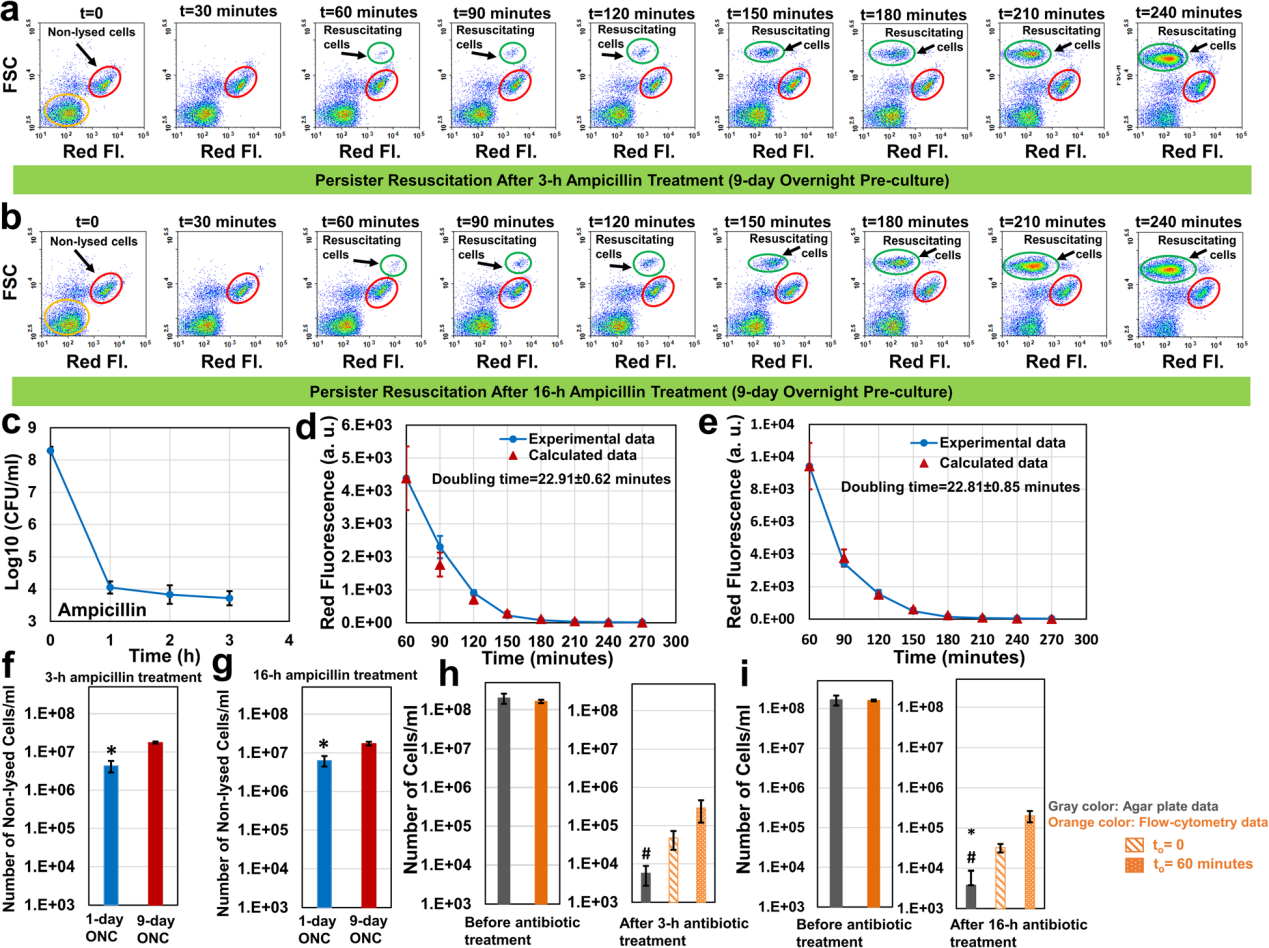
The effect of long-term pre-culturing on persister resuscitation. **a**-**b** Cells from 9-day overnight pre-cultures were inoculated in fresh media, cultured until OD_600_ = 0.25, and then treated with ampicillin for 3 h or 16 h. After ampicillin treatments, cells were transferred to LB broth to monitor persister resuscitation with a flow-cytometry (*N* = 4). **c** Cells during the antibiotic treatments were plated to obtain the bi-phasic kill curve (*N* = 4). **d** The doubling time of the resuscitating cells after 3-h ampicillin treatments (*N* = 4). **e** The doubling time of the resuscitating cells after 16-h ampicillin treatments (*N* = 4). **f**-**g** Comparison of non-lysed cell levels in short-term (1 day) and long-term (9 days) pre-cultures (*N* = 4). * indicates a significant difference between short-term and long-term pre-cultures (*P* < 0.05). **h**-**i** Resuscitating cell levels after 3- and 16-h antibiotic treatments (*N* = 4). * indicates a significant difference between flow-cytometry (t_o_ = 0) and agar plate cell counts (*P* < 0.05). # indicates a significant difference between flow-cytometry (t_o_ = 60 min) and agar plate cell counts (*P* < 0.05). Error bars represent the standard deviations. ONC: Overnight pre-culture

Persister cell populations are heterogeneous and exhibit diverse, but poorly characterized metabolic and gene expression activities. Whereas some persisters can arise from growing cell subpopulations [8], many persister cells are found to be at the non-proliferating state before antibiotic treatments, and they are largely formed by passage through the stationary phase [18]. To analyze the effects of stationary-phase lengths on persister resuscitation, we cultured the overnight pre-cultures up to 9 days. On certain days (1, 2, 3, 5, 7, and 9 days), cells from overnight pre-cultures were transferred to fresh media, cultured until they reached the mid-exponential growth phase (OD_600_ = 0.25), and then treated with ampicillin for 3 h or 16 h. Although our results clearly showed that long-term pre-culturing did not significantly impact the persister resuscitation and doubling time (Fig. 4a,b,d,e and Additional file 1: Fig. S5ABCD and Fig. S6ABCD), it significantly increased the non-lysed cell (VBNCs) levels (Fig. 4f,g). Approximately, 10% percent of exponential phase cells obtained from 9-day overnight pre-cultures were not lysed by ampicillin (Fig. 4f,g). We note that mCherry levels of the non-lysed cells from long-term pre-cultures are lower than those from short-term pre-cultures (Figs. 2a vs. 4a); this is because of the increased mCherry protein degradation and/or leakage in long-term pre-cultures (Additional file 1: Fig. S7), known characteristics of the late-stationary phase cells [19, 20]. Interestingly, we observed a significant difference between persister levels obtained from flow-cytometry diagrams and CFU measurements (Fig. 4h,i and Additional file 1: Fig. S2CD). Our analysis indicates that at least 10-fold more cells (based on t_o_ = 0 estimates from flow-cytometry analysis, Fig. 4h,i) resuscitated in liquid cultures compared to solid media. This interesting phenomenon, which has been observed in both 3-h- and 16-h-ampicillin-treated cultures (Fig. 4h,i), indicates the importance of culture conditions to persister cell resuscitation.

**Fig. 5:**
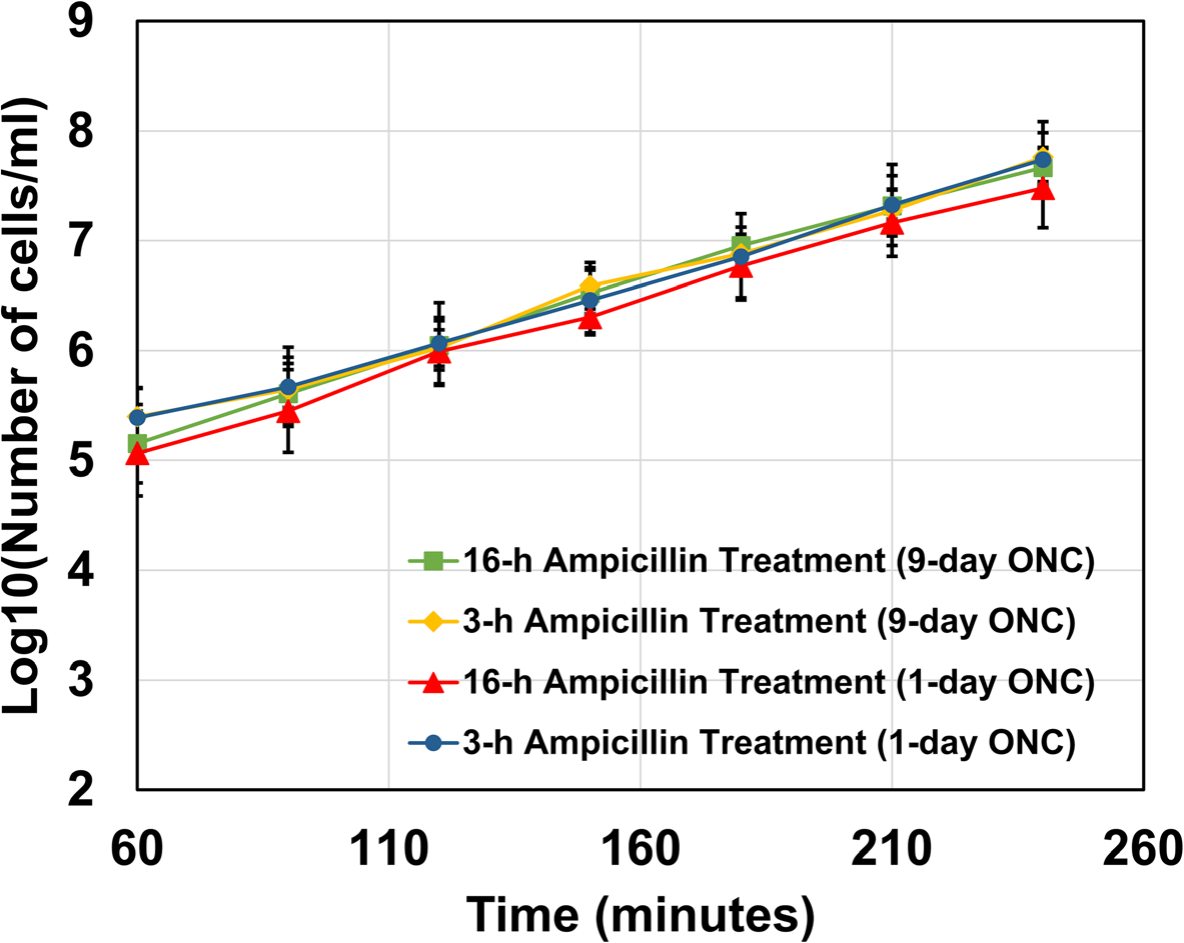
Growth curves of resuscitating cells in liquid cultures. The number of resuscitating cells at indicated time points was quantified with a flow cytometer (*N* = 4). Error bars represent the standard deviations. ONC: Overnight pre-culture

Stationary-phase lengths in pre-cultures significantly affected the ability of persister cells to wake up in solid media. Persisters levels (as measured by CFU counts on agar plates) in long-term pre-cultures were found to be almost 50-fold less than those identified in short-term pre-cultures (Figs. 2c vs. 4c). However, this was not observed in liquid media. In fact, our flow cytometry analysis showed that a similar amount of persister cells started to wake up within 1 hour in liquid cultures, regardless of the pre-culturing lengths or ampicillin treatment times (persister data highlighted with patterned orange columns in Figs. 2e, 3d and 4hi). The cell growth profiles of the resuscitating cells in liquid cultures were also found to be very similar in all conditions tested (Fig. 5). Doubling times obtained from these growth profiles (Fig. 5) were found to be consistent with those obtained from mCherry-dilution method (Additional file 1: Table S1). Overall, these results indicate that although long-term culturing does not significantly affect the persister resuscitation and doubling time in liquid cultures, it impacts the non-lysed cell levels. Our study also clearly shows that the culture environment affects the ability of non-lysed cells to wake-up.

### Ampicillin cannot lyse the pre-proliferating cells in the presence of arsenate

**Fig. 6:**
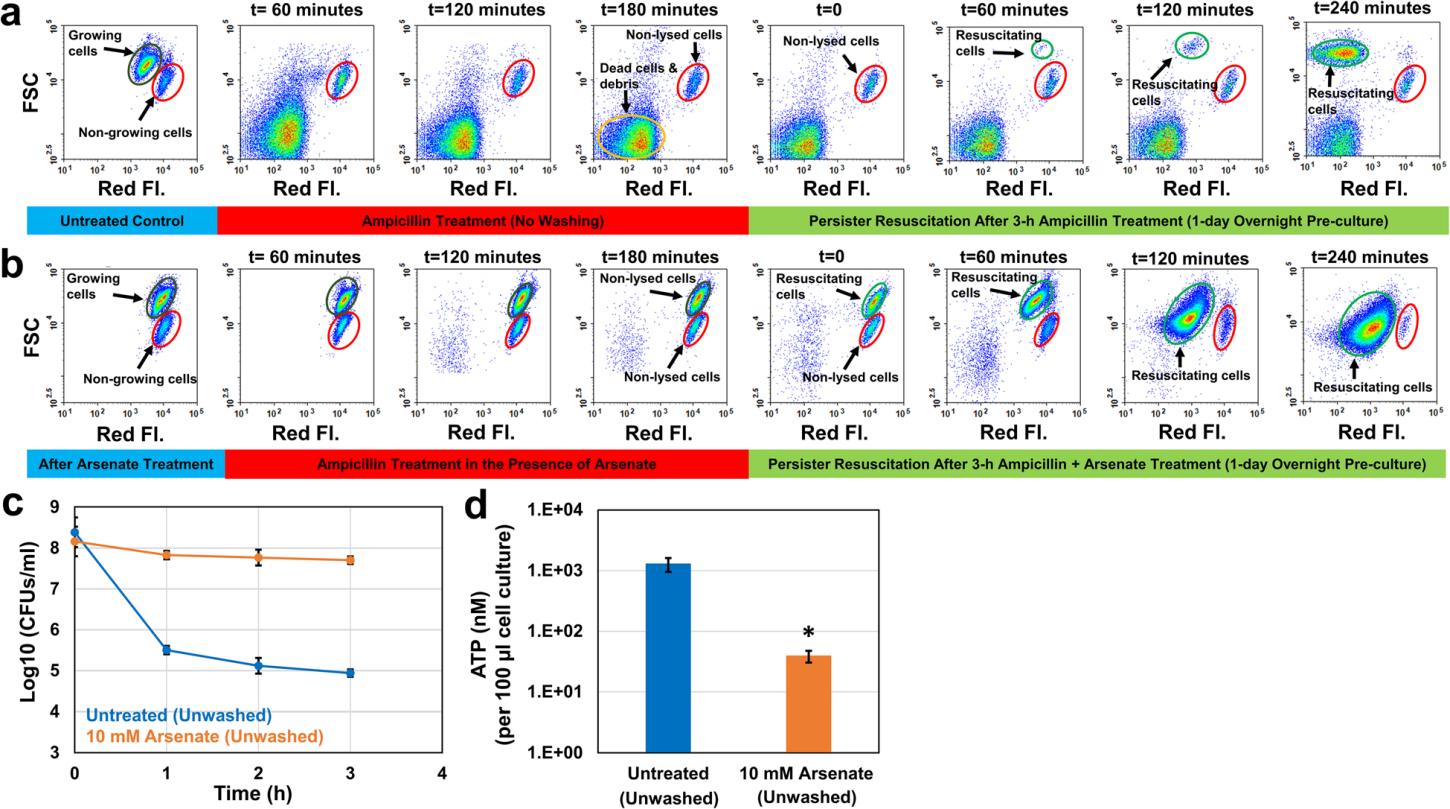
The effects of arsenate and ampicillin co-treatment on persister formation, ATP levels, and persister resuscitation. **a**-**b** Exponentially growing cells (OD_600_ = 0.25) were treated with solvent (DI water) or 10 mM arsenate for 30 min. At t = 180 min, ampicillin was added into cell cultures. After the ampicillin treatment, cells were transferred to fresh LB media to monitor persister resuscitation with a flow cytometry. **c** Cells during the antibiotic treatments were plated to generate the kill curves (*N* = 3). **d** ATP levels of control and co-treatment groups were measured before ampicillin treatment using a BacTiter-Glo™ Microbial Cell Viability Assay kit (*N* = 3). *indicates a significant difference between control and co-treatment groups (*P* < 0.05)

A correlation between ATP depletion and persistence has been consistently shown in previous studies using arsenate-treated cell cultures [21–23]. Arsenate, which competes with phosphate [24], is thought to increase persistence in bacteria by reducing ATP production [21, 22]. To elucidate the resuscitation characteristics of cell populations exhibiting increased persistence, we treated the exponential-phase cultures (t = 150 min, OD_600_ = 0.25) with 10 mM arsenate for 30 min followed by ampicillin treatment with arsenate (co-treatment), as described previously [21, 22]. Thirty-min pretreatment was found to be sufficient to inhibit cell proliferation (Fig. 6b, the cell subpopulations highlighted with dark-green circles) and to reduce ATP levels (Fig. 6d), consistent with the studies published by Kim Lewis’ group [21–23]. As expected, the persister levels of arsenate-treated cultures were significantly higher than those of the control groups (Fig. 6c). However, our flow cytometry analysis indicates that this increase in persistence may be due to the bacteriostatic effect of arsenate during antibiotic treatment, as ampicillin was not able to lyse the pre-proliferating cells in the presence of arsenate (Fig. 6b, the cell subpopulations highlighted with dark-green circles). Interestingly, all these non-lysed, pre-proliferating cells started to resuscitate within an hour in fresh media after the removal of ampicillin and arsenate (Fig. 6b, the cell subpopulations highlighted with light-green circles), and reached the stationary phase very quickly (Fig. 6b, t = 240 min and Additional file 1: Fig. S8). We note that the cultures at t = 240 min were further diluted for flow-cytometry analysis; this explains the reduction in the number of non-growing cells shown in the flow diagram (Fig. 6b, t = 240 min and Additional file 1: Fig. S8). Due to their transition to the stationary phase, FSC of resuscitating cells decreased at t = 240 min (Fig. 6b). This is expected as the reductive division resulting in small spherical cells is known to take place when cells enter the stationary phase [19]. We also note that the chromosomal mCherry expression cassette is slightly leaky during the stationary phase even in the absence of IPTG; therefore, mCherry levels of resuscitated cells at t = 240 min (Fig. 6b) are slightly higher than expected. This might be due to the up-regulation of CRP/cAMP complex during the stationary phase, as this complex tightly regulates the lac promoters. However, this phenomenon does not impact our current analysis; therefore, we are not planning to investigate this further. The control group has undergone similar procedures without receiving any arsenate treatment. All proliferating cells were lysed by ampicillin, and only a small fraction of intact cells from the non-growing cell subpopulation was able to resuscitate in the control group (Fig. 6a), consistent with our aforementioned results.

**Fig. 7:**
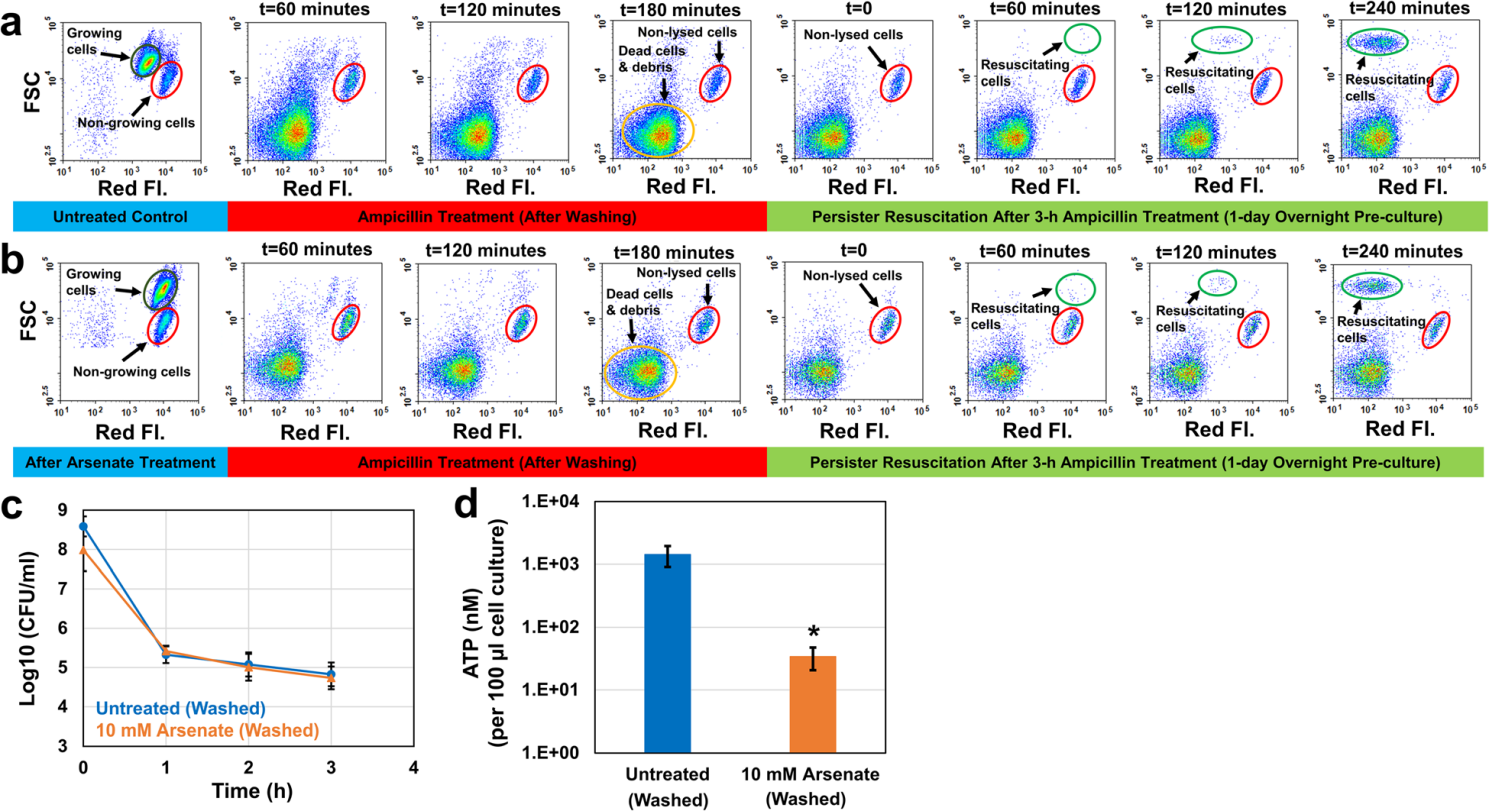
The effects of arsenate pretreatment on persister formation, ATP levels, and persister resuscitation. **a**-**b** Exponentially growing cells (OD_600_ = 0.25) were treated with solvent or 10 mM arsenate for 30 min. At t = 180 min, cells were first washed to remove the arsenate and then resuspended in fresh media with ampicillin (without arsenate). After the ampicillin treatment, cells were transferred to fresh LB media to monitor persister resuscitation. **c** Cells during the antibiotic treatments were plated to generate the kill curves (*N* = 3). **d** ATP levels of control and pretreatment groups (*N* = 3). *indicates a significant difference between control and pretreatment groups (*P* < 0.05)

Antibiotic treatments have been performed in the presence of arsenate in previous studies [21, 22], which makes it ambiguous whether the arsenate-induced persistence is indeed due to the ATP depletion. To figure out this, we performed the persister assays mentioned above in the absence of arsenate. After the pretreatment with arsenate, we washed the cells with Phosphate Buffered Saline (PBS) solution to remove the chemical and transferred the washed cells to fresh media with ampicillin. This washing procedure did not affect the exponential-phase persister (Figs. 6c vs. 7c) and ATP levels (Figs. 6d vs. 7d) in control groups. Our flow cytometry analysis showed that growing subpopulations in both pretreatment and control groups were lysed within 3 h by ampicillin (Fig. 7a,b). Unexpectedly, both groups have similar persister levels (Fig. 7c), despite the significant reduction in ATP levels observed in arsenate-pretreated cultures (Fig. 7d). Also, persisters from non-growing cell subpopulations started to resuscitate within an hour in both pretreatment and control groups when they were exposed to fresh media (Fig. 7a,b). Overall, these results indicate that the ATP-depleted cultures do not necessarily exhibit increased persistence (Fig. 7c); in fact, the enhanced persistence shown in Fig. 6c is potentially due to the synergistic effect of arsenate in co-treated cultures.

## Discussion

In this study, we presented an exploratory methodology, incorporating the flow cytometer with cells expressing a fluorescent indicator of cell division, to monitor resuscitating cells after antibiotic treatments. We used ampicillin for our assays, which is commonly used to isolate persister populations [10, 12], given its ability to lyse the proliferating cells [15, 16]. We have demonstrated that only a small fraction of non-lysed cell populations wakes up, and this phenomenon has been observed within 1 hour (independent of pre-culturing and treatment times) after transferring the antibiotic-treated cells to fresh liquid media.

Using a microfluidic device, Windels and coworkers have shown that persister resuscitation is stochastic and resuscitation time ranges from a few minutes to 13 h, with 30–40% cells dividing within 1 hour [25]. We note that culture conditions, treatment times and the antibiotic used in our study are significantly different than those in the study of Windels and coworkers. Here, we transferred antibiotic-treated cells to fresh, rich media in baffled flasks and cultured with shaking. These culture conditions are known to promote cell growth [26, 27]. It is possible that the rapid overpopulation of progenies originating from the early-dividing cells might have prevented the growth of other resuscitating cells emerging at later time points. This might also explain why the non-growing cells characterized by lower FSC in arsenate treated cultures shown in Fig. 6b did not go through the same resuscitation process as in the other conditions. Unfortunately, growing cells were not lysed by ampicillin in the presence of arsenate and these cells reached the stationary phase quickly upon their resuscitation after the removal of the chemicals (Fig. 6b).

Recurrent and chronic infections are generally associated with biofilms where persister cells are significantly enriched and evade the host immune system. Although some persistent infections are associated with clinically apparent-chronic symptoms, some cases are asymptomatic for a long time [28]. Given that antibiotics can diffuse through biofilms [29], the concept of a “protected niche” is not enough to explain the prolonged survival of microorganisms in the human body [28]. In fact, this can be explained by the “adaptive plasticity” of bacteria, i.e. the ability of bacteria to “play dead”, suggested by Walsh McDermott in 1958 [30]. This hypothesis has been supported by many studies clearly demonstrating that bacteria causing recurrent infections can be present within the host in a non-replicating or slowly replicating state that cannot be easily cultured in vitro [31]. Lleo and coworkers reported that 14% of clinical samples from 182 patients, which did not form bacterial colonies on standard media, had VBNCs [32]. Since both VBNCs and persisters are concurrently present in cell cultures [12] and capable of continued pathogenesis in host organisms [28], the simultaneous characterization of these two phenotypes is essential. Unfortunately, studies characterizing persister cell physiology rarely focused on VBNC cells. A few research groups have already verified that there are many more VBNC cells than persisters in antibiotic-treated cultures [8, 9]. Using live/dead staining and water-soluble tetrazolium salt dye, we demonstrated that these VBNC cells are indeed live cells with the ability to efficiently metabolize certain carbon sources [12].

In our study, we showed that long-term (9 days) pre cultures have ~ 3–4 fold more VBNC cells compared to short-term (1 day) pre-cultures. We note that stresses associated with stationary-phase cultures, such as nutrient starvation, oxidative stress, pH change and toxic waste from starved cells are already known to induce VBNC cell formation [31, 33–36]. Another interesting observation is related to resuscitating cell levels determined from the standard plating method and flow-cytometry analysis. Although antibiotic treatment or pre-culturing times have minimal effects on persister wake-up and doubling times in liquid cultures, the number of persisters resuscitating on agar plates was found to be significantly lower than that of liquid cultures when we used 9-day pre-cultures. The culturability of VBNC cells is already known to depend on the culture conditions [37]. Reduced resuscitation of VBNC cells can be observed on agar media compared to liquid cultures due to the impurity of agar, cell density and restricted diffusion of nutrients [37]. The oxidative stress on agar media was also shown to inhibit the resuscitation of VBNC cells [14].

Energy metabolism was shown to be inversely correlated with persistence in previous studies [21–23, 38–40], where exponential-phase cultures were generally treated with a metabolic inhibitor, such as arsenate. Shan et al. have shown that the reduction in intracellular ATP concentration led to the inactivation of the antibiotic target site [22]. They treated exponentially growing *E. coli* cells with arsenate for half an hour followed by ampicillin treatment and observed a significant increase in persister levels [22]. Braetz et al. have found a similar mechanism in *Salmonella* strains [40]. They reported a significant increase in ciprofloxacin persisters in arsenate-treated cell cultures [40]. Unfortunately, persister assays in these studies were performed in the presence of arsenate, which may not eliminate the synergistic effects of co-treatments.

## Conclusions

In summary, we presented here a methodology that aims to monitor persister resuscitation. This method also enables us to quantify non-resuscitating cells (VBNCs), which are generally ignored by the persister research community, despite certain phenotypic similarities between these cells and persisters. We believe that this approach will provide a platform to study persistence and VBNC state that can be expanded to different antibiotics and organisms. This experimental approach can also be used to isolate resuscitating cells using cell sorting instruments to directly assess their physiological characteristics with conventional methodologies (e.g., genomics, proteomics, and metabolomics) to fill a fundamental knowledge gap within our understanding of persister physiology.

## Methods

### Bacterial strains and plasmids

*Escherichia coli* MG1655 MO strain and pQE-80 L plasmids harboring genes encoding a green fluorescent protein (GFP) were previously generated [8, 41, 42]. *E. coli* MO strain harbors a chromosomally integrated IPTG-inducible *mCherry* expression cassette [8, 42], which is used to monitor cell proliferation at the single cell level. pQE-80 L expression system has a kanamycin resistance gene (*kan*^*R*^) with an IPTG-inducible synthetic *T5* promoter and a strong constitutive *LacI*^*q*^ promoter as a repressor, to tightly regulate the expression of GFP [41].

### Media, chemicals, and culture conditions

All chemicals were purchased from Fisher Scientific (Atlanta, GA), VWR International (Pittsburg, PA) or Sigma Aldrich (St. Louis, MO). LB liquid media prepared from its components (5 g yeast extract, 10 g tryptone and 10 g sodium chloride in 1 L ultra-pure DI water) [41, 43]. LB agar media (40 g premixed LB agar in 1 L ultra-pure DI water) was used to enumerate CFUs. Cells were washed with PBS solution to remove the antibiotics. For persister assays, 60 μg/ml (10X of MIC) ampicillin was used. To determine the MIC of ampicillin, cells were cultured in 2 ml LB media in 14 ml round bottom falcon tubes where the antibiotic was serially diluted (2-fold) [43, 44]. The MIC range of ampicillin for *E. coli* MG1655 MO is 3.125–6.25 μg/ml [43]. Kanamycin (50 μg/ml) was added to the culture media for the selection and retention of plasmids. IPTG (1 mM) was used to induce mCherry and GFP expression [41]. Overnight pre-cultures were prepared by inoculating frozen cells (− 80 °C) in 2 ml LB broth in a 14 ml round bottom falcon tube and grown for 24 h or more (up to 9 days) at 37 °C with shaking (250 rpm). IPTG (1 mM) was added if the expression of fluorescent proteins was required.

### Persister assays

Overnight pre-cultures of *E. coli* MG1655 MO cells were diluted 100-fold into 25 ml LB media in 250-ml baffled flasks and grown in the presence of 1 mM IPTG (to induce mCherry) at 37 °C with shaking (250 rpm). We note that, depending on the overnight pre-culture lengths, the inoculation rates were adjusted to transfer the same amount of pre-culture cells to the media. When the cell density reached OD_600_ = 0.25 at t = 150 min (cell count ~ 10^8^ cells/ml), cultures were treated with ampicillin at 37 °C with shaking. At designated time points, t = 0 (right before the antibiotic treatment), 1, 2, 3 and 16 h, 1 ml samples were collected and centrifuged at 13,300 rpm for 3 min to pellet the cells. After centrifugation, 950 μl of supernatant was removed from each microcentrifuge tube, and 950 μl of PBS was then added to resuspend the cells. This procedure was repeated at least twice to dilute the antibiotics to sub-MIC levels. After washing, cells were serially diluted in PBS using round-bottom 96-well plates, and, then, 10 μl of cell suspensions were spotted on LB agar. The agar plates were incubated at 37 °C for 16 h to enumerate the CFUs. We note that new colonies were not formed when incubated beyond 16 h.

### Monitoring persister resuscitation with a flow cytometer

Entire cultures of *E. coli* MG1655 MO cells, treated with ampicillin as described above, were collected at indicated time points (3 h or 16 h) and pelleted in 50 ml falcon tubes by centrifuging at 4700 rpm for 15 min. The pelleted cells were then suspended in 1 ml PBS and transferred to microcentrifuge tubes. After washing with PBS to remove the antibiotic and IPTG completely, the cells were inoculated in 25 ml fresh LB media in 250 ml baffled flasks and incubated at 37 °C with shaking in the absence of IPTG. Every 30 min, 200 μl cell cultures were collected from the flasks and diluted in 800 μl PBS solution and immediately analyzed with a flow cytometer to measure the mCherry levels of resuscitating cells (NovoCyte Flow Cytometer, NovoCyte 3000RYB, ACEA Biosciences Inc., San Diego, CA).

To verify that resuscitating cells are live and capable of expressing proteins, we used *E. coli* MG1655 strain with pQE-80 L plasmids harboring IPTG inducible *gfp*. The cells were grown and treated with ampicillin as described above but without IPTG. The inducer (1 mM) was added after transferring the antibiotic-treated cells to fresh media. Resuscitating cells expressing GFP was monitored with the flow cytometer. Cells carrying empty vectors served as a negative control. All samples were assayed with lasers emitting at 488 nm for GFP or 561 nm for mCherry. Fluorescence was collected by 530/30 nm bandpass filter for GFP and 615/20 nm bandpass filter for mCherry. In each run, we measured 60,000 events. Sterile PBS, wild-type (WT) *E. coli*, mCherry-positive and GFP-positive cells were used to gate the cell populations on flow-cytometry diagrams. Cells were counted using the volumetric-based cell counting feature of the NovoCyte Flow Cytometer; this enabled us to calculate the growing, non-growing, non-lysed or resuscitating cell levels in cultures after taking dilution factors into consideration.

### Persister cell growth and doubling time quantification

In the absence of IPTG, mCherry concentration in growing cells was reduced with time due to the cell division. Because of this correlation, the red fluorescence reduction with the culture time was used to calculate the cell-doubling time with the following fluorescence decay equation:

$$ F={F}_0{2}^{-\left(t-{t}_o\right)/{t}_d} $$

where F_0_ is the mean fluorescence intensity of the resuscitating cell population at t_o_; t_d_ is the doubling time; and F is the mean fluorescence intensity of resuscitating cell population at time t. Fluorescent measurements of dividing cells were performed with a flow cytometer as described above. Since we were able to measure the mean fluorescence intensity of resuscitating cells around 60 min after transferring the antibiotic-treated cells to fresh media, t_o_ was chosen to be 60 min. Excel SOLVER was used to calculate doubling time by minimizing the sum of normalized mean square errors (NMSE) between experimental and predicted model data.

To calculate the initial number of persister cells that resuscitated, the classical doubling time formula for the exponential-growth phase was used:

$$ N={N}_o{2}^{\left(t-{t}_o\right)/{t}_d} $$

where N_o_ is the initial number of cells that started to resuscitate at t_o_; t_d_ is the doubling time of that particular experimental condition and calculated from the fluorescence decay equation as described above. N is the number of resuscitated cells that were counted at time t. The flow-cytometry cell counts obtained at t = 240 min were used for N. N_o_ levels were calculated from the exponential growth equation using two different scenarios: t_o_ = 0 and t_o_ = 60 min.

### Arsenate pretreatment

Overnight pre-cultures of *E. coli* MG1655 MO cells were diluted 100-fold into 25 ml LB media in 250-ml baffled flasks and grown as described above. When the cell density reached OD_600_ = 0.25 (t = 150 min), cultures were treated with 10 mM arsenate (sodium hydrogen arsenate heptahydrate, Fisher Scientific, catalog# AAA1827522) for 30 min at 37 °C with shaking (250 rpm). At t = 180 min, the arsenate-pretreated cells were challenged with ampicillin (60 μg/ml, 10X of MIC) for 3 h. Persister quantification at designated time points was performed as described above. We note that, when necessary, cells were washed with PBS to remove the arsenate and suspended into 25 ml LB media in 250-ml baffled flasks followed by the ampicillin treatments. Control groups, treated with solvent only (DI water), have undergone the same procedures.

### ATP measurements

The intracellular ATP levels of control and treatment (arsenate) groups were measured using a BacTiter-Glo™ Microbial Cell Viability Assay kit (Catalog# G8230, Promega Corporation, Madison, WI) according to manufacturer’s guideline. Fresh LB media was used to measure the background luminescence. Standard curves were generated by measuring the luminescence of ATP solutions at known concentrations. These solutions were prepared by serially diluting rATP (Promega catalog# P1132) in fresh LB.

### Statistical analysis

In this study, at least three independent biological replicates were performed for each condition. Two-tailed student t-test with unequal variance was used to determine statistical significance where the threshold *P*-value is 0.05 [41]. All t_d_ or N_o_ calculations were performed for each biological replicate. Each experimental or computational data point in the figures represents mean value ± standard deviation.

## Supplementary information

**Additional file 1: Fig. S1.** Cell growth of *E. coli* MG1655 MO strain in LB broth medium. **(A)** mCherry positive cells from 1-day overnight pre-cultures were diluted 100-fold in fresh media and grown in the absence of IPTG. At designated time points, samples were collected to monitor the proliferating cells with a flow cytometer (*N* = 4). **(B)** The doubling time of the resuscitating cells was calculated using the decay equation (see the Materials and Methods) and the mean fluorescence intensities of dividing cells (highlighted with dark-green circles) (*N* = 4). **Fig. S2.** The comparison of calculated and experimentally quantified resuscitating cell levels. **(A)** Cells from overnight (1 day) pre-cultures were diluted (100-fold) in fresh media, cultured to mid-exponential phase (OD600 = 0.25) and then treated with ampicillin (60 μg/ml) for 3 h. After the treatment, cells were transferred to fresh media and cultured to count the resuscitating cells at t = 60 min with a flow cytometer. The resuscitating cells were also calculated using the classical exponential growth equation as described in the main text. **(B)** The similar experimental procedures were performed as described in A. Cells were obtained from 1-day overnight pre-cultures; however, persister assays were performed for 16 h. **(C)** Cells were obtained from 9-day overnight pre-cultures, and persister assays were performed for 3 h. **(D)** Cells were obtained from 9-day overnight pre-cultures, and persister assays were performed for 16 h. **Fig. S3.** Monitoring persister resuscitation with a GFP expression system. **(A-B)** Stationary-phase cultures of *E. coli* cells harboring pQE-80 L empty vector (EV) or pQE-80 L::*gfp* were inoculated (1:100) into fresh media and cultured without IPTG. Upon reaching the mid-exponential growth phase (OD600 = 0.25), cells were treated with 60 μg/ml ampicillin for 3 h. Cells were then washed to remove the antibiotic and resuspended in fresh media with IPTG. At designated time points samples were collected and analyzed with a flow cytometry. As expected, resuscitating cells (highlighted with green circles), exhibiting higher forward side scatter (FCS) signals due to the elongation characteristic of growing cells, were able to express GFP. **(C)** Resuscitating-cell growth was monitored by measuring the GFP positive cells with a flow cytometer. It was also monitored using the mCherry dilution method as described in Fig. 2 in the main text. Although we did not observe a significant difference between the results of these two methods, over-expressing GFP with high-copy plasmids slightly reduced cell growth, an expected observation. Green Fl.: Green Fluorescence. **Fig. S4.** VBNC levels in short- and long-term ampicillin treatments. Viable but non-culturable (VBNC) cells were determined by subtracting the number of persisters (agar plate data) from non-growing cells (flow cytometry data) (*N* = 4). **Fig. S5.** The effect of long-term pre-culturing on persister resuscitation. To test the stationary-phase lengths on persister resuscitation, overnight pre-cultures were cultured up to 9 days. On certain days (A: 2-day, B: 3-day, C: 5-day, and D: 7-day), cells were inoculated (1:100-fold) in fresh media in the presence of IPTG and cultured until the mid-exponential-growth phase (OD600 = 0.25) was obtained. Then, cells were treated with 60 μg/ml ampicillin. Three hours after the treatment, cells were transferred to fresh media to monitor persister resuscitation at indicated time points. Note that the data corresponding to 9-day overnight cultures was provided in the main text. **Fig. S6.** The effect of long-term pre-culturing on persister resuscitation. Cells were grown the same way as described in Fig. S5; however, the ampicillin treatment was performed for 16 h. A: 2-day, B: 3-day, C: 5-day, and D: 7-day. **Fig. S7.** mCherry levels in the stationary phase cells obtained from 1-day or 9-day overnight pre-cultures. mCherry levels were measured with a flow cytometer (*N* = 4). Protein degradation/leakage is known to be observed in long-term cultures. **Fig. S8.** Resuscitation of cells after arsenate and ampicillin treatment. Arsenate and ampicillin treated cells were washed and inoculated in fresh LB media to monitor the persister resuscitation. Samples were analyzed with flow cytometer every 30 min (*N* = 3). Table S1. Estimating the doubling times of resuscitating cells using the cell counts or mCherry dilution method. ONC=Overnight pre-culture.

## Data Availability

Data provided in this paper including supplementary materials are sufficient to assess the findings of this paper. Additional data of this paper can be obtained upon request.

## Abbreviations

VBNC: Viable but non-culturable
ATP: Adenosine triphosphate
IPTG: Isopropyl β-D-1-thiogalactopyranoside
FSC: Forward scatter
OD6_00_: Optical density at 600 nm wavelength
CFU: Colony forming unit
GFP: Green fluorescence protein
cAMP: Cyclic adenosine monophosphate
CRP: cAMP receptor protein
PBS: Phosphate buffered saline
MIC: Minimum inhibitory concentration
NMSE: Normalized mean square errors
Kan: Kanamycin
