# Supplementary material for: Flow-cytometry analysis reveals persister resuscitation characteristics
Source: BMC Microbiol. 2020 Jul 8;20:202. doi: 10.1186/s12866-020-01888-3 (PMC7346475; doi:10.1186/s12866-020-01888-3)
Supplement: Supplementary file 1 — Additional file 1: Fig. S1. Cell growth of E. coli MG1655 MO strain in LB broth medium. (A) mCherry positive cells from 1-day overnight pre-cultures were diluted 100-fold in fresh media and grown in the absence of IPTG. At designated time points, samples were collected to monitor the proliferating cells with a flow cytometer (N = 4). (B) The doubling time of the resuscitating cells was calculated using the decay equation (see the Materials and Methods) and the mean fluorescence intensities of dividing cells (highlighted with dark-green circles) (N = 4). Fig. S2. The comparison of calculated and experimentally quantified resuscitating cell levels. (A) Cells from overnight (1 day) pre-cultures were diluted (100-fold) in fresh media, cultured to mid-exponential phase (OD600 = 0.25) and then treated with ampicillin (60 μg/ml) for 3 h. After the treatment, cells were transferred to fresh media and cultured to count the resuscitating cells at t = 60 min with a flow cytometer. The resuscitating cells were also calculated using the classical exponential growth equation as described in the main text. (B) The similar experimental procedures were performed as described in A. Cells were obtained from 1-day overnight pre-cultures; however, persister assays were performed for 16 h. (C) Cells were obtained from 9-day overnight pre-cultures, and persister assays were performed for 3 h. (D) Cells were obtained from 9-day overnight pre-cultures, and persister assays were performed for 16 h. Fig. S3. Monitoring persister resuscitation with a GFP expression system. (A-B) Stationary-phase cultures of E. coli cells harboring pQE-80 L empty vector (EV) or pQE-80 L::gfp were inoculated (1:100) into fresh media and cultured without IPTG. Upon reaching the mid-exponential growth phase (OD600 = 0.25), cells were treated with 60 μg/ml ampicillin for 3 h. Cells were then washed to remove the antibiotic and resuspended in fresh media with IPTG. At designated time points samples were collected an [file 12866_2020_1888_MOESM1_ESM.pdf]

Supplementary Information

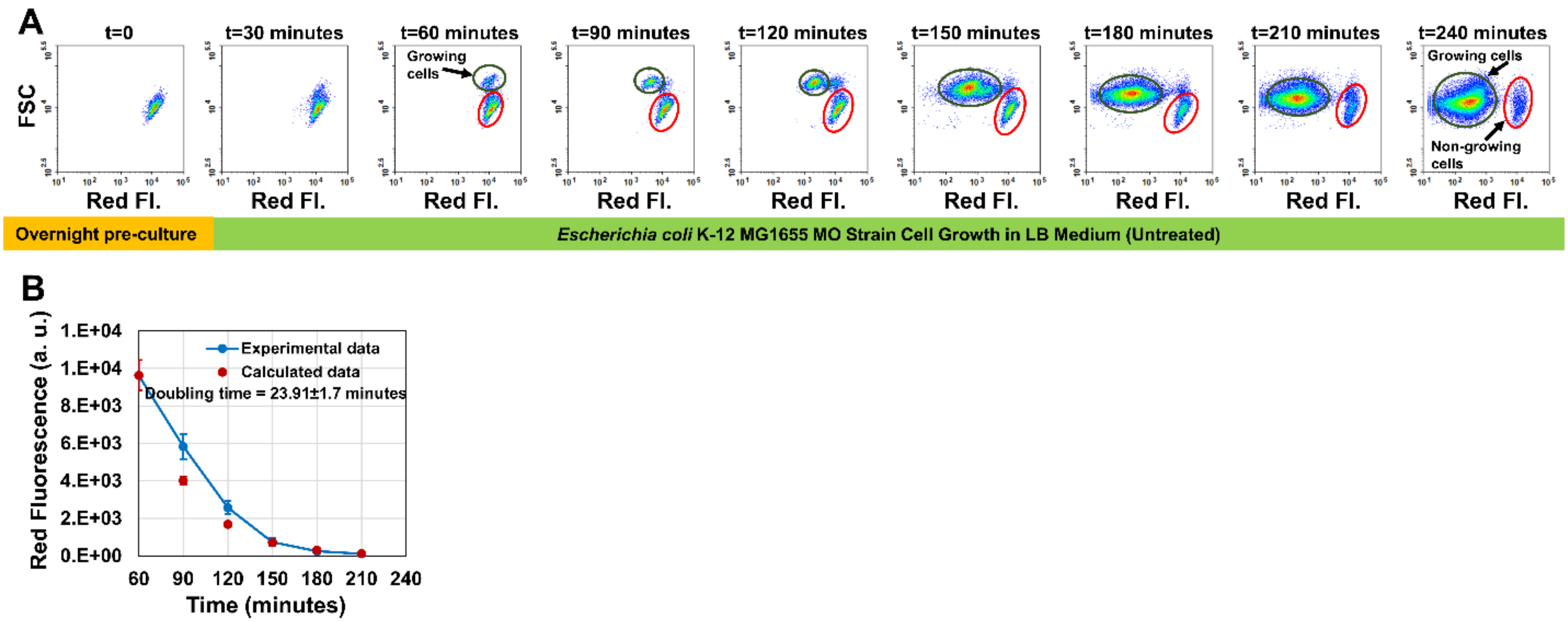

Fig. S1. Cell growth of *E. coli* MG1655 MO strain in LB broth medium. (A) mCherry positive cells from 1-day overnight pre-cultures were diluted 100-fold in fresh media and grown in the absence of IPTG. At designated time points, samples were collected to monitor the proliferating cells with a flow cytometer (N=4). **(B)** The doubling time of the resuscitating cells was calculated using the decay equation (see the Materials and Methods) and the mean fluorescence intensities of dividing cells (highlighted with dark-green circles) (N=4).

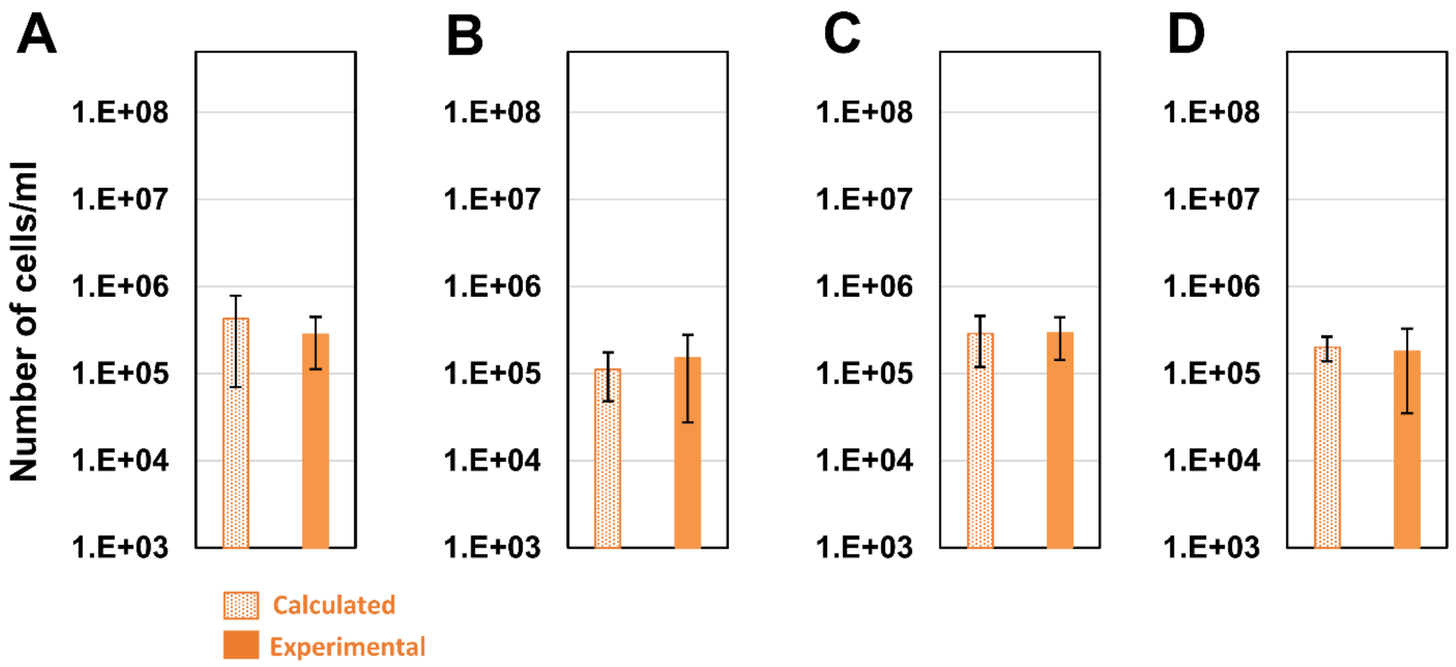

Fig. S2. The comparison of calculated and experimentally quantified resuscitating cell levels. (A) Cells from overnight (1 day) pre-cultures were diluted (100-fold) in fresh media, cultured to mid-exponential phase (OD_{600}=0.25) and then treated with ampicillin (60 µg/ml) for 3 h. After the treatment, cells were transferred to fresh media and cultured to count the resuscitating cells at t=60 minutes with a flow cytometer. The resuscitating cells were also calculated using the classical exponential growth equation as described in the main text. **(B)** The similar experimental procedures were performed as described in A. Cells were obtained from 1-day overnight pre-cultures; however, persister assays were performed for 16 h. **(C)** Cells were obtained from 9-day overnight pre-cultures, and persister assays were performed for 3 h. **(D)** Cells were obtained from 9-day overnight pre-cultures, and persister assays were performed for 16 h.

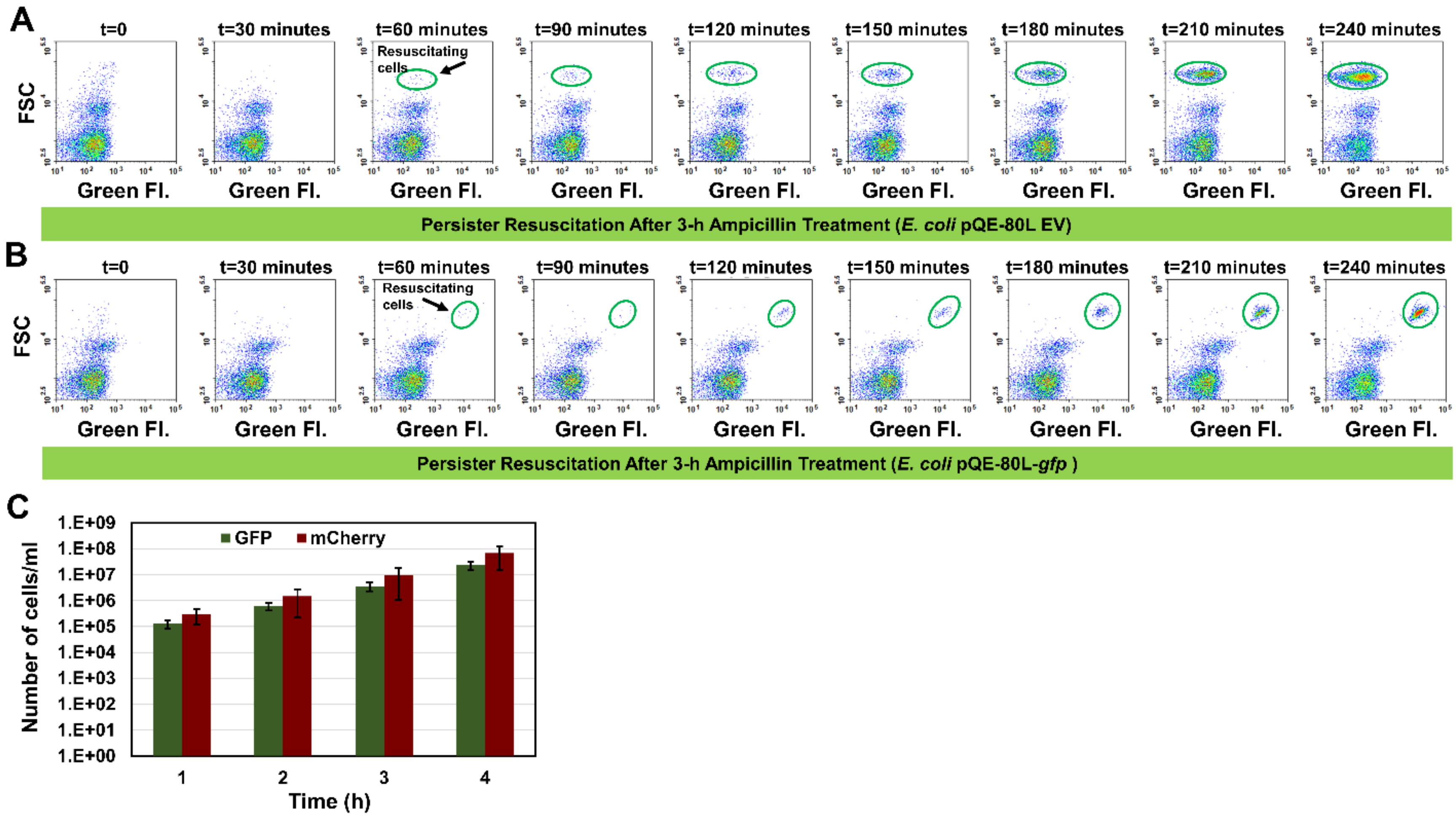

Fig. S3. Monitoring persister resuscitation with a GFP expression system. (A-B) Stationary-phase cultures of *E. coli* cells harboring pQE-80L empty vector (EV) or pQE-80L::*gfp* were inoculated (1:100) into fresh media and cultured without IPTG. Upon reaching the mid-exponential growth phase (OD_{600}=0.25), cells were treated with 60 µg/ml ampicillin for 3 h. Cells were then washed to remove the antibiotic and resuspended in fresh media with IPTG. At designated time points samples were collected and analyzed with a flow

cytometry. As expected, resuscitating cells (highlighted with green circles), exhibiting higher forward side scatter (FCS) signals due to the elongation characteristic of growing cells, were able to express GFP. **(C)** Resuscitating-cell growth was monitored by measuring the GFP positive cells with a flow cytometer. It was also monitored using the mCherry dilution method as described in Fig. 2 in the main text. Although we did not observe a significant difference between the results of these two methods, over-expressing GFP with high-copy plasmids slightly reduced cell growth, an expected observation. Green Fl.: Green Fluorescence.

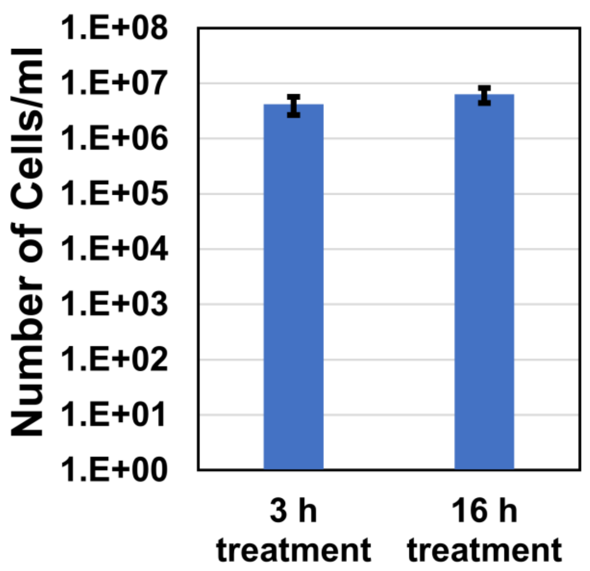

Fig. S4. VBNC levels in short- and long-term ampicillin treatments. Viable but non-culturable (VBNC) cells were determined by subtracting the number of persisters (agar plate data) from non-growing cells (flow cytometry data) (N=4).

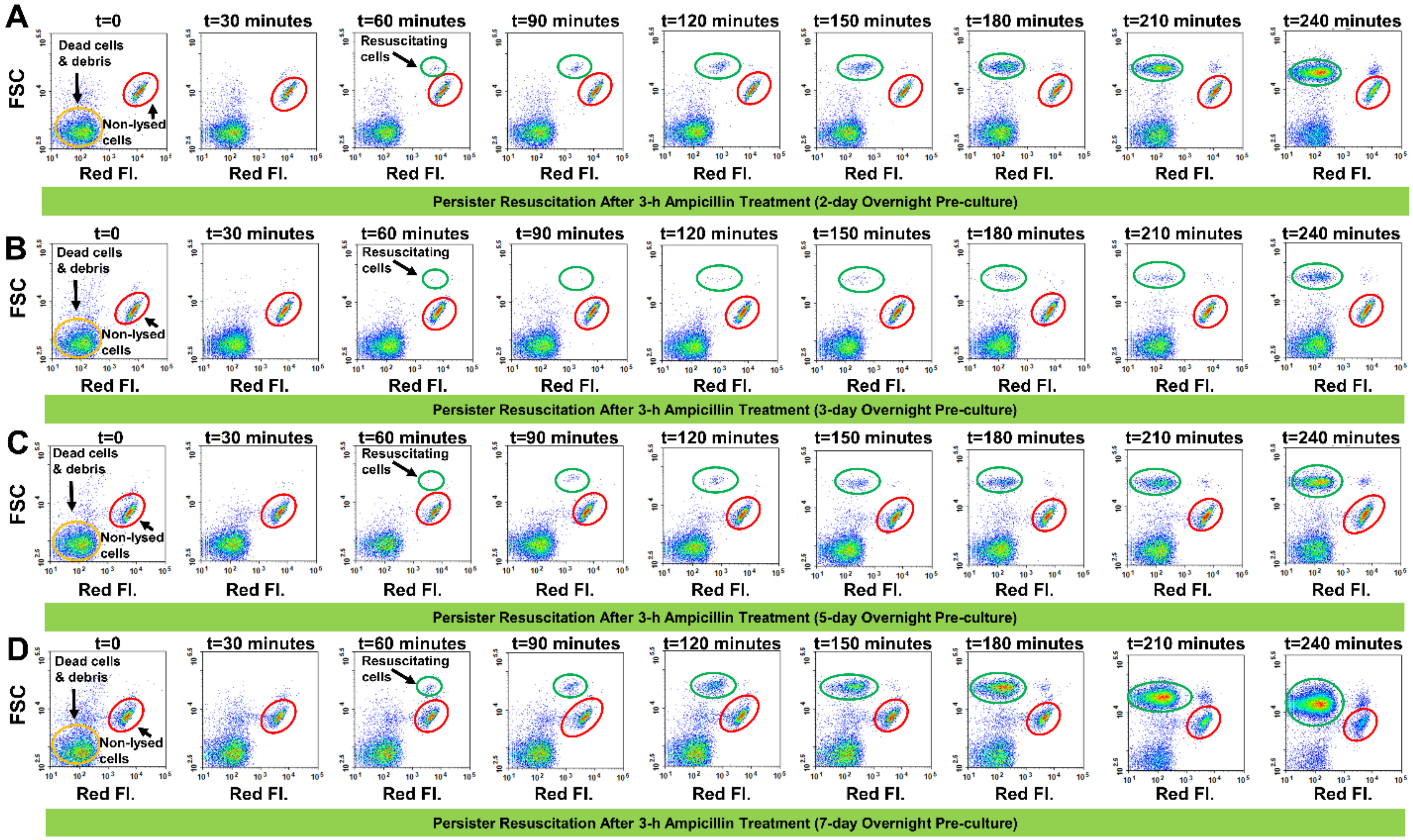

Fig. S5. The effect of long-term pre-culturing on persister resuscitation. To test the stationary-phase lengths on persister resuscitation, overnight pre-cultures were cultured up to 9 days. On certain days (A: 2-day, B: 3-day, C: 5-day, and D: 7-day), cells were inoculated (1:100-fold) in fresh media in the presence of IPTG and cultured until the mid-exponential-growth phase (OD_{600}= 0.25) was

obtained. Then, cells were treated with 60 µg/ml ampicillin. Three hours after the treatment, cells were transferred to fresh media to monitor persister resuscitation at indicated time points. Note that the data corresponding to 9-day overnight cultures was provided in the main text.

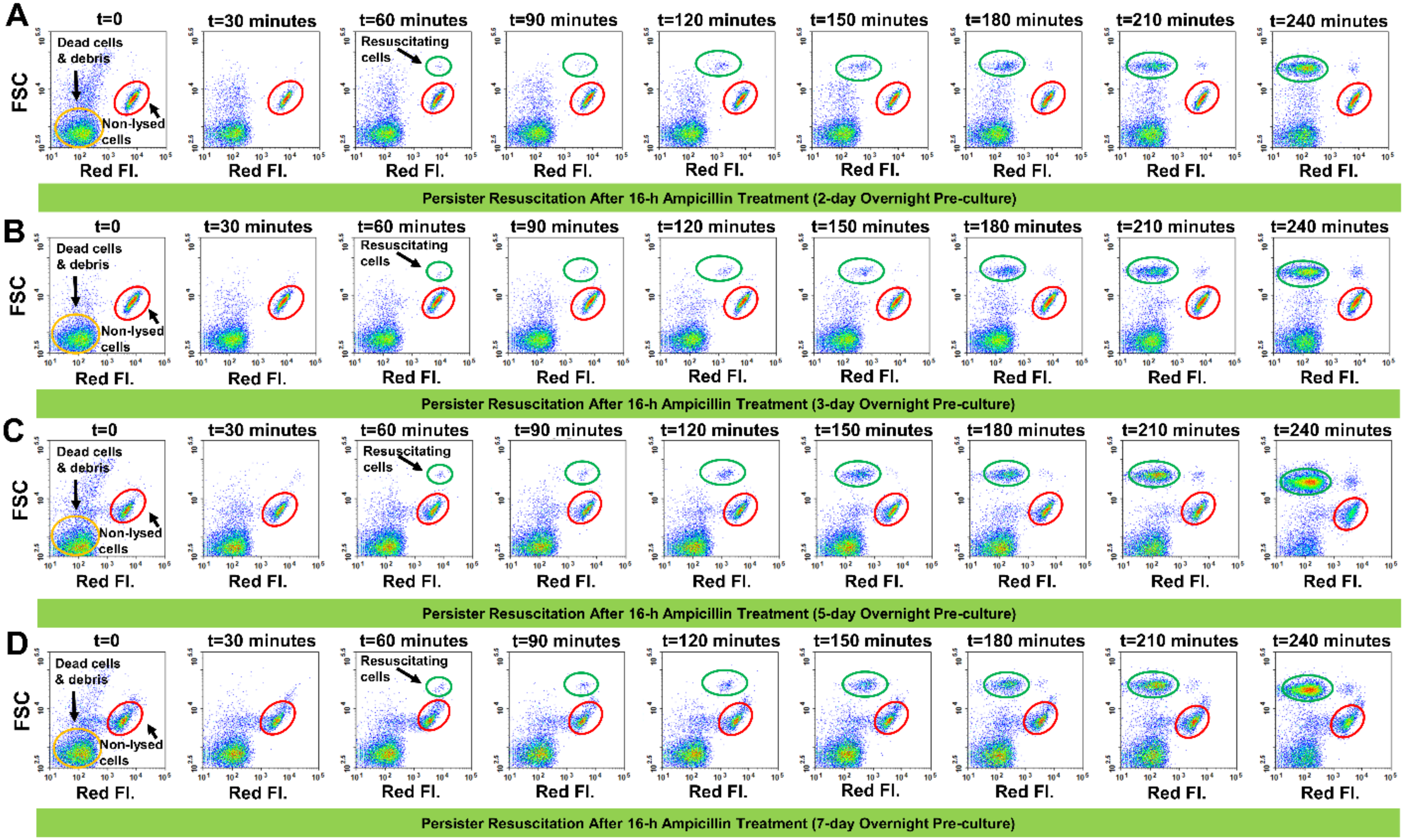

Fig. S6. The effect of long-term pre-culturing on persister resuscitation. Cells were grown the same way as described in Fig. S5; however, the ampicillin treatment was performed for 16 h. A: 2-day, B: 3-day, C: 5-day, and D: 7-day.

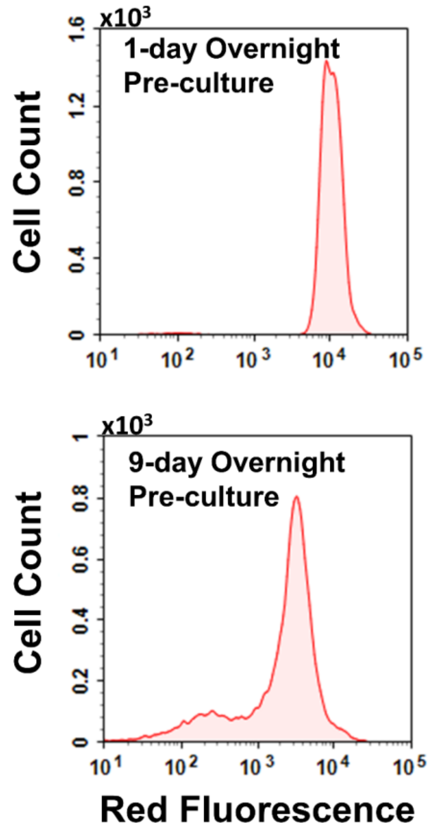

Fig. S7. mCherry levels in the stationary phase cells obtained from 1-day or 9-day overnight pre-cultures. mCherry levels were measured with a flow cytometer (N=4). Protein degradation/leakage is known to be observed in long-term cultures.

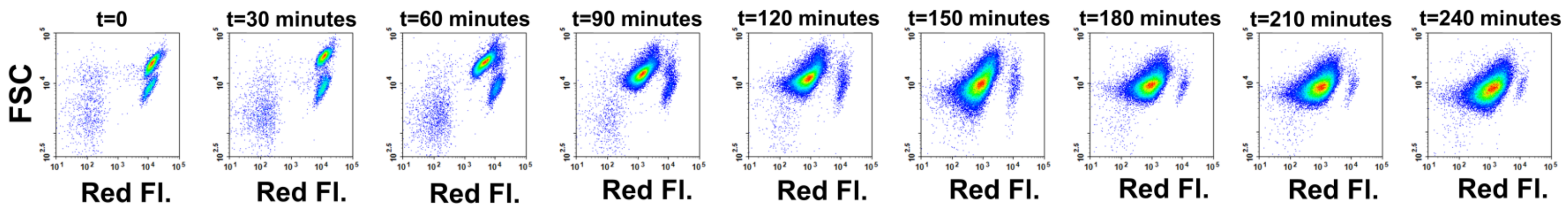

Fig. S8. Resuscitation of cells after arsenate and ampicillin treatment. Arsenate and ampicillin treated cells were washed and inoculated in fresh LB media to monitor the persister resuscitation. Samples were analyzed with flow cytometer every 30 minutes (N=3).

Table S1. Estimating the doubling times of resuscitating cells using the cell counts or mCherry dilution method. ONC=Overnight pre-culture.

Doubling time model based on cell counts						
	1st Replicate	2nd Replicate	3rd Replicate	4th Replicate		
	Doubling time, td	Doubling time, td	Doubling time, td	Doubling time, td	Average	Standard dev.
24h ONC – 3h Treatment	21.99400403	22.59760267	22.12473165	21.74232361	22.11466549	0.358946287
24h ONC- 16h Treatment	25.28765919	23.27802839	22.40419125	21.06553651	23.00885384	1.77082917
9days ONC - 3h Treatment	21.83786178	21.85523561	21.42719663	22.61891132	21.93480134	0.497124062
9days ONC - 16h Treatment	22.23173542	23.54339675	21.27433876	21.71917893	22.19216247	0.982091966

Doubling time model based on red fluorescence						
	1st Replicate	2nd Replicate	3rd Replicate	4th Replicate		
	Doubling time, td	Doubling time, td	Doubling time, td	Doubling time, td	Average	Standard dev.
24h ONC - 3h Treatment	21.29094393	21.05210874	26.24060475	24.60356735	23.2968062	2.545304484
24h ONC- 16h Treatment	21.24121183	21.44421747	21.28139869	21.76024566	21.43176841	0.235920291
9days ONC - 3h Treatment	22.14114091	23.21434537	22.7297443	23.57360036	22.91470774	0.620897017
9days ONC - 16h Treatment	22.42561399	21.88912094	23.85241099	23.06892473	22.80901766	0.846449384
